# Interleukin-6 and pulmonary hypertension: from physiopathology to therapy

**DOI:** 10.3389/fimmu.2023.1181987

**Published:** 2023-06-28

**Authors:** Wei-Jie Xu, Qiong Wu, Wen-Ni He, Shang Wang, Ya-Lin Zhao, Jun-Xia Huang, Xue-Shen Yan, Rong Jiang

**Affiliations:** ^1^Department of Clinical Laboratory, Shanghai Pulmonary Hospital, Tongji University School of Medicine, Shanghai, China; ^2^Department of Pulmonary and Critical Care Medicine, Tongren Hospital, Shanghai Jiao Tong University School of Medicine, Shanghai, China; ^3^Department of Cardiopulmonary Circulation, Shanghai Pulmonary Hospital, School of Medicine, Tongji University, Shanghai, China; ^4^Department of Respiratory Critical Care Medicine, The First Hospital of Kunming, Kunming, China; ^5^Department of Hematology, Affiliated Hospital of Qingdao University, Qingdao, Shandong, China

**Keywords:** pulmonary hypertension, interleukin-6, signaling pathway, inflammation, nuclear factor kappa-B

## Abstract

Pulmonary hypertension (PH) is a progressive, pulmonary vascular disease with high morbidity and mortality. Unfortunately, the pathogenesis of PH is complex and remains unclear. Existing studies have suggested that inflammatory factors are key factors in PH. Interleukin-6 (IL-6) is a multifunctional cytokine that plays a crucial role in the regulation of the immune system. Current studies reveal that IL-6 is elevated in the serum of patients with PH and it is negatively correlated with lung function in those patients. Since IL-6 is one of the most important mediators in the pathogenesis of inflammation in PH, signaling mechanisms targeting IL-6 may become therapeutic targets for this disease. In this review, we detailed the potential role of IL-6 in accelerating PH process and the specific mechanisms and signaling pathways. We also summarized the current drugs targeting these inflammatory pathways to treat PH. We hope that this study will provide a more theoretical basis for targeted treatment in patients with PH in the future.

## 1 Introduction

Pulmonary hypertension (PH) is a serious progressive pulmonary vascular disease, the main pathological feature of which is pulmonary artery disease associated with increased intrapulmonary arterial pressure, luminal coarctation, and progressive increase in pulmonary vascular resistance (PVR) due to remodeling of small pulmonary vessels, and in severe cases, right heart failure (RHF) and death (1–3). Patients with PH mainly present with progressively deteriorating RHF, and early clinical symptoms such as dyspnea, chest pain, chest tightness, and shortness of breath lack specificity; however, with the progression of the disease, irreversible decline in cardiopulmonary function can be life-threatening.

The pathogenesis of PH is complex and is thought to be the result of a combination of genetics (gene mutations), epigenetics (DNA methylation, histone modification, microRNA [miRNA], etc.) and environmental factors (hypoxia, inflammation, drugs, oxidative stress, etc.), but it has not yet been understood (4, 5). The current research on the pathogenesis of PH mainly focuses on exploring the roles of the above factors in the physiological processes of pulmonary artery endothelial cell dysfunction (PAEC), pulmonary artery smooth muscle cell (PASMC) remodeling and proliferation (6). Some studies suggest that the inflammatory response and inflammatory pathways play important roles in the pathogenesis of PH (7). A large number of inflammatory cells (such as macrophages, lymphocytes, neutrophils, etc.) infiltrated around pulmonary artery blood vessels were observed in PH clinical patients and PH animal models (8). Studies have demonstrated that macrophages around pulmonary vessels can be activated by fibroblasts and subsequently enhance inflammatory pathways such as interleukin-6 (IL-6) signal transduction (9), thereby promoting blood PH vascular reconstruction, suggesting that the onset of PH may be related to a variety of inflammatory cells and inflammatory factors. Therefore, targeted immune inflammation therapy may become a new direction of PH treatment (7).

Perivascular inflammation is a prominent feature in the pathogenesis of PH, but the exact role of the inflammatory pathway remains controversial. How do the inflammatory cells impact the proliferation of endothelial cells, smooth muscle cells and fibroblasts? A large number of studies have confirmed the role of IL-6 in the pathogenesis of PH. The lung function in patients with PH is inversely correlated with elevated serum IL-6 concentrations (10). A similar observation was displayed in PH mouse model (11). The inflammatory mechanism involved in IL-6 may become a potential target for PH therapy. Therefore, the purpose of this review aims to summarize the role of IL-6 in PH pathogenesis and potential regimes.

IL-6 was first discovered in 1980 by Weissenbach (12). It consists of 184 amino acids forming a peptide chain, with a molecular weight between 21,000-30,000 due to differences in glycosylation modifications and phosphorylation of protein peptides (13). IL-6 is a small molecular glycoprotein encoded by chromosome 7p15-21, which consists of four α structures and usually exists in monomer form (14). At the beginning of its discovery, IL-6 was named hybridoma/plasmacytoma growth factor, B-cell differentiation factor, B-cell stimulation factor 2, cytotoxic T-cell differentiation factor and heterocyst stimulating factor.

IL-6 is the most widespread multipotent cytokine and can be secreted by T cells, B cells, monocyte-macrophages, dendritic cells, mast cells, fibroblasts, smooth muscle cells, endothelial cells, osteoblasts, glomerular mesangial cells and tumor cells in the human body (15) and play roles in immune regulation, inflammation, hyperplasia, metabolism, and regeneration (16). In healthy bodies, IL-6 levels at physiological concentrations help regulate inflammation; however, in the pathological state, the increase in plasma IL-6 concentration lead to pathological damage (10).

### 1.1 Evidence of IL-6 in patients with PH

The general purpose of the clinical classification of PH remains to categorize clinical conditions associated with PH, based on similar pathophysiological mechanisms, clinical presentation, haemodynamic characteristics, and therapeutic management, including Group 1 PAH, Group 2 PH associated with left heart diseases, Group 3 PH associated with lung diseases and/or hypoxia, Group 4 PH associated with pulmonary artery obstructions and Group 5 PH with unclear and/or multifactorial mechanisms (17).

The relationship between IL-6 and PH has been explored for more than 27 years. In 1995, Humbert et al. first studied the serum levels of proinflammatory factors in a patient with severe Group 3 PH secondary to chronic obstructive pulmonary disease (COPD) and normal healthy people, and found that the serum IL-6 concentration of patients was higher than that in the control group (20 ± 14 pg/mL vs. 6 ± 0 pg/mL, p < 0.01), suggesting that IL-6 may be an important inflammatory mediator of PH (9).

First, IL-6 can be used as a biomarker for the assessment of PH severity. IL-6 is associated with the severity and pathological progression of Group 3 PH in patients with COPD and is an independent risk factor for pathogenesis (18, 19). The serum levels of growth factors vascular endothelial growth factor (VEGF), platelet-derived growth factor-B (PDGF-B), and transforming growth factor-β1 (TGF-β1), as well as IL-6, were significantly higher in PH patients compared with controls and that elevated IL-6 concentrations were independently correlated with mortality in patients with PH, and that IL-6 may be a biomarker for PH prognosis (20). Simpson et al. demonstrated that elevated blood concentrations of IL-6 are associated with pulmonary vascular remodeling in patients with PH; IL-6 may be released by PASMCs, serum IL-6 is associated with specific clinical phenotypes and outcomes in patients with PH, and IL-6 may become a potential therapeutic target for early disease diagnostic markers and treatment of PAH, including connective tissue diseases associated PAH (CTD-PAH) (21). Another study showed that the serum of IL-6 was significantly elevated in patients with idiopathic and hereditary PAH (IPAH and HPAH), and that IL-6 as a prognosis predictor was better than traditional predictors such as six-minute walking distance and hemodynamics in Group 1 PAH patients (22). Interestingly, serum IL-6 levels have been found to be significantly associated with hemodynamic deterioration and clinical deterioration (death, transplantation, palliative surgery) but are associated with a reduced incidence of pediatric IPAH, possibly because the child’s immune system is not fully established (23).

When the severity of pulmonary vascular lesions in Group 1 PAH patients was comparable, IL-6 levels were independently negatively correlated with right ventricular (RV) dysfunction (24, 25). Elevated IL-6 levels can predict mortality and were associated with Group 1 PAH patient survival (20, 21, 26). Prins and colleagues found that serum IL-6 levels in patients with PH were independently correlated with RV function and RV- pulmonary artery (PA) coupling. Although pulmonary vascular disease is comparable in patients with Group 1 PAH, patients with higher serum IL-6 concentrations have more severe RV dysfunction and reduced RV-PA coupling (27).

Since increase of IL-6 was documented in the pathogenesis of Group 1 PAH patients, inhibition of IL-6 signal transduction may provide a new therapeutic approach for patients with PH. The IL-6R antagonist tocilizumab were able to block IL-6 immune inflammation regulation, thereby effectively reducing pulmonary artery pressure, which could reduce adverse outcomes and improve the quality of life in PH patients. This suggests that the inhibition of IL-6 mediated pathways may be a promising strategies for PH (28).

In addition, single nucleotide polymorphisms in the IL-6 promoter region can transcribe and regulate the expression of IL-6, and populations with special IL-6 gene polymorphism exhibit a higher risk of Group 1 PAH and a more severe phenotype. It was shown that IPAH risk was related to the (-572C/G) [rs1800796] polymorphism in the IL-6 promoter region but not to the -6331T/C [rs10499563] polymorphism (29). Similarly, Chaouat and colleagues observed that elevated serum IL-6 levels in patients with Group 3 PH were suggestive of an association with mean pulmonary artery pressure (mPAP), but not in COPD patients without PH. The IL-6 GG genotype has a higher mPAP and is related with more severe PH than the CG/CC genotype (30).

### 1.2 Evidence of IL-6 in the PH animal model

Many animal studies have shown that the cytokine IL-6 plays a role in driving the pathogenesis of PH. In mouse animal models, IL-6 deletion blocks the inflammatory process of PH. IL-6-deficient mice had better right ventricular (RV) function than wild-type mice. Immunostaining showed less hypoxia-induced recruitment of lung inflammatory cells in IL-6-deficient mouse, but there was no change in the expression of adhesion molecules (intercellular cell adhesion molecule-1 [ICAM-1] and vascular cell adhesion molecule-1 [VACM-1]) or cytokines (monocyte chemoattractant protein-1 [MCP-1]) (11). Similarly, IL-6 is produced by PASMCs and the medial layer of the pulmonary arteries of mice under chronic hypoxia, while IL-6 knockout mice do not develop the effects of hypoxia-induced PH (31). This indicates that the absence of IL-6 may prevent the PH process. Interestingly, Maston and others discovered that IL-6 may be produced and promote pulmonary artery cell migration through the trans-signaling pathway (that is, IL-6 combines with soluble IL-6 receptor [SIL-6R] released by activated T cells to promote pulmonary artery cell migration and chronic hypoxia-induced PH (31) (**Table 1**).

**Table 1:** Role of drugs associated IL-6 in pulmonary hypertension.

Drug	Intervention	Model	Mechanism and Outcome
Resveratrol (trans 3,5,4’-trihydroxystilbene) (32)	Stilbenoid polyphenol	HPAECs; HPASMCs; MCT-PH rat; SuHx rat	↓NF-κB, ↓BMP/SMAD↓, ERK1/MAPK, ↓PI3K/AKT →↓IL-6, ↓IL-8, ↓IL-10, ↓IL-18, ↓TNF-α, ↓TGF-β, ↓MCP-1, ↓PDGF →↓HPASMC proliferation, ↓PAR, ↓RVSP
GS-444217 (33)	ASK1 inhibitor	Rat cardiomyocytes; MCT-PAH; SuHx rat; Murine of RV pressure overload induced by PAB	↓p38 MAPK, ↓JNK↓ASK1 →↓IL-6, ↓TIMP-1, ↓CXCL-1, ↓CXCL3, ↓TGF-β →↓fibroblasts activation and migration →↓PAR, ↓RVR, ↓RVH
Ruxolitinib (34)	Jak1/Jak2 inhibitor	HPASMCs (IPAH); MCT-PH; SuHx rat	↓Jak2/Stat3→↓IL-6 →↓HPASMC proliferation and migration →↓PAR, ↓mPAP, ↓RVSP, ↓RVH
FGF21 (35, 36)	Member of FGF19	PASMC; HPAECs(hypoxia-exposed); SuHx rat	↓miR-27b→↑PPARγ→↓NF-κB →↓IL-6, ↓IL-1β, ↓TNF-α →↓mPAP, ↓PAR, ↓RVH
T4 (37)	MIF inhibitor	HPASMCs; Macrophage (RAW 264.7) SuHx rat	↓MIF activation →↓NF-κB, ↓ERK/MAPK, ↑CXCR2 mRNA →↓ANG-2, ↓IL-6→↓HPASMC proliferation
18 β-GA (38)	NF-κB inhibitor	HPASMCs (PDGF-B-induced); MCT-PH rat	↑IκB, ↓ERK/eIF2α/NF-κBp65 →↓IL-6, ↓TNF-α, MCP-1, ↓GRP78 →↓ERS, ↓HPASMC proliferation and DNA synthesis →↓PAR, ↓mPAP, ↓RVH, ↓RVSP
PF (39)	Monoterpene glycoside	PASMC (PDGF-B-induced); MCT-PH rat	↓TAK1, ↑BMPR-II→↓MAPK/NF-κB →↓IL-6, ↓IL-1β, ↓TNF-α →↓EndMT, ↓HPAEC proliferation and migration, ↑HPAEC apoptosis →↓PAR, ↓RVR, ↓RVSP
Luteolin (40)	Flavonoid compound	PASMC (PDGF-B induced); MCT-PH rat	↓LATS1, ↓YAP, ↓YAP nuclear localization →↓PI3K/AKT →↓PASMC proliferation and migration →↓PAR, ↓RVH, ↓RVSP
Baicalin (41)	TNF-α inhibitor	HPASMC (TNF-α induced); MCT-PH rat	↓NF-κB, ↓IL-6, ↓IL-1β, ↓TNF-α →↑BMPR-II →↑smad1/5/8, ↑Id1 →↓PASMC proliferation and migration →↓RVH, ↓RVSP
Isorhamnetin (42)	BMPR-II agonist	HPASMC (TNF-α induced); MCT-PAH rat	↑BMPR-II→↑smad1/5→↓IL-6, ↓TNF-α, ↑Id1/3 →↓HPASMCs proliferation →↓PAR, ↓mPAP, ↓RVH, ↓RVSP
Telmisartan (43)	PPARγ agonist	HPAECs; MCT-PH rat	↑PPARγ→↑PI3K/Akt→↑eNOS→↑NO→↓PAR, ↓RVSP
ANDRO (44)	Anti-inflammatory agent	HPASMCs (PAH); SuHx rat	↓TLR4/NF-κB→↑BMPR-II, ↓[Ca^2+^], ↓IL-6, ↓IL-8, ↓ET-1, ↓VEGF, ↓NOXs/Nrf2 →↓ROS →↓Akt, ↓ERK/MAPK, ↓JNK/MAPK, ↑p38/MAPK, ↓HPASMC proliferation, ↑HPASMC apoptosis →↓PAR, ↓RVSP, ↓RVH
Saquinavir, glycyrrhizn; TAK-242 (45)	HMGB1 inhibitors; TLR4 inhibitors	PASMCs; SuHx rat	↓HMGB1/TLR4→↓IL-6, ↓IL-1β, ↓TNF-α, ↑BMPR-II →↑Smad1/5/8, ↑Id1, IL-6 →↓PASMC proliferation and migration →↓PAR, ↓RVSP, ↓RVH
Tempol (46)	Superoxide dismutase mimetic	HPASMCs (BMPR-II mutation); Mouse with BMPR-II (+/-)	↓STAT3→↓IL-6→↓PAR, ↓RVSP
SBT (47)	Traditional Tibetan medicinal formula	MCT-PH rat; SuHx rat	↓NF-κB, ↓MAPK→↓IL-6, ↓IL-1β, ↓TNF-α →↓mPAP, ↓PAR
PBA (48)	ERS inhibitor	MCT-PH rat	↓ERs→↓IL-6, ↓TNF-α, ↓MMP-2, ↓MMP-9, ↓RV cardiomyocytes apoptosis →↓mPAP, ↓RVR, ↓RVH, ↓RVSP
DA (49)	SGLT2 inhibitor	MCT-PH rat	↓TLR4, ↓NF-κBp65 →↓potassium channel, ↓L-type Ca channel↓ →↓VA, ↓ventricular hypertrophy and fibrosis
CIHH (50)	Physical therapeutics	MCT-PH rat	↓NF-κB, ↓p38/MAPK →↓IL-6, ↓TNF-α, ↓CD4+ T cells, ↑CD8+ T cells →↓pulmonary artery cell proliferation →↓mPAP, ↓PAR, ↓RVH
Thymoquinone and Sevoflurane (51)	Pro apoptotic and migration inhibitor;	MCT-PH rat	↓p38/MAPK/NF-κB, ↓Bax/Bcl-2, ↓MMP-2 →PASMC proliferation and migration →↓PAR, ↓RVH, ↓RVSP
Sevoflurane (52)	Anesthetic	MCT-PH rat	↓ERK1/MAPK, ↓ERK2/MAPK, ↓P38/MAPK, ↓NF-κB, ↓IκBα →↓IL-6, ↓TNF-α →↓PAR, ↓RVR, ↓RVH, ↑RVF
Baicalein (53)	Natural flavonoid	MCT-PH rat	↓p38/MAPK, ↓ERK/MAPK, ↓JNK/MAPK, ↓NF-κB →↓IL-6, ↓IL-1β, ↓TNF-α, ↓BAX, ↓Caspase-3, ↑Bcl-2 →↓apoptosis, ↓PAR, ↓RVR, ↓RVH, ↓RVSP, ↑RVF
Capsaicin (54)	SP depleting	MCT-PH rat	↓SP→↓p38/MAPK →↓IL-6, ↓IL-1β, ↓TNF-α →↓PAR, ↓RVSP, ↓RVH, ↑RVF
Aspirin (55)	ERK/MAPK inhibitor	MCT-PH rat	↓ERK1/MAPK, ↓ERK2/MAPK→↑eNOS →↓endothelial damage, →↓PAR, ↓RVSP, ↓RVH
MTEP (56)	mGluR5 inhibitor	MCT-PH rat	↓mGluR5, ↓P38/MAPK, ↓PI3K/AKT →↓IL-6, ↓TNF-α, ↓Ang 2, ↓VEGF →↓RVR, ↓RVSP
Masitinib (57)	Tyrosine kinase inhibitor	MCT-PH rat	↓ERK/MAPK, ↓CXCR4/CXCL12, ↓PDE-5 mRNA, ↓PDGF-B mRNA, ↓C-KIT, ↓PDGF-B, ↑cGMP →↓PAR, ↓ RVH, ↓RVSP
Tanshinone IIA, Genistein (58, 59)	PI3K agonist	MCT-PH rat	↑PI3K/Akt→↑eNOS →↑NO→↓mPAP, ↓PAR, ↓RVSP, ↓RVH
Dexamethasone (60)	BMPR-II Inducer	MCT-PH rat	↑BMPR-II→↓IL-6→↓PASMC proliferation→↓PAR, ↓RVH, ↓RVSP
Glycoprotein 130 Inhibitor (61)	Glycoprotein 130 Inhibitor	MCT-PH rat	↑BMPR-II→↓IL-6, ↓STAT3 →↓ PASMC proliferation, ↑PASMCs’ apoptosis →↓PAR, ↓mPAP, ↓RVSP
STS (62)	PI3K inhibitor	SuHx rat	↓PI3K/AKT/mTOR →↓IL-6, ↓IL-8, ↓ TNF-α, ↓Bcl-2, ↑Bax →↑apoptosis, ↑autophagy→↓PAP, ↓RVH
Atorvastatin (63, 64)	PI3K agonist Notch1 inhibitor	SuHx rat	↑PI3K/AKT, ↓Notch1 →↓IL-6, ↓TNF-α, ↓MPO, ↓Bcl-2, ↑Caspase-3 →↑lung tissues apoptosis→↓RVSP, ↓RVH
Sildenafil (65)	PDE-5 inhibitor	SuHx rat	↓Notch3, ↓Hes1→↓PASMC proliferation, ↑PASMC apoptosis→↓PAP, ↓RVH
Baicalin (66)	PPARγ agonist	SuHx rat	↑PPARγ, ↓HO1→↓HMGB1, ↓RAGE →↓IL6, ↓TGF-β1→↓PAP, ↓RVH
EP (67)		PASMCs (hypoxia -induced); Anastomosis of the common carotid and jugular veins-induced PH mouse	↓HMGB1, ↓RAGE →↓HPASMCs proliferation →↓PAR, ↓RVH
AA (68)	Notch inhibitor	RAW 264.7 macrophages (LPS-induced): Experimental sepsis mouse	↓Notch3, ↓DLL4, ↓Hes1, ↓IκB, ↓NF-Kb →↓IL-6, ↓IL-1β, ↑NO
Glycyrrhizic (69)	Saponin triterpenoid	3-NP-induced HD rats	↓HMGB1/TLR4/NF-κB →↓IL-6, ↓TNF-α, ↓caspase-3, ↑Bcl-2,
Hesperidin (70)	Notch signaling pathway inhibitor	RSV-induced bronchiolitis mouse	↓Jagged1, ↓Notch1→↓IL-6, IL-4↓, ↓TNF-α, ↑IL-10↑, ↑M2 macrophage polarization →lung tissues damage
Atorvastatin (71)	NF-κB inhibitor	HPASMCs (LPS induced)	↓ CRP→↓NF-κB→↓ IL-6, ↓MCP-1
Prostanoid (72)	Camp-mobilising	HUVECs	↑cAMP→↑SOCS3, ↑PKA →vasodilation, ↓IL-6→↓Jak/Stat→↓PAR

AA, asiatic acid; ANDRO, andrographolide; ANG-2, angiopoietin-2; ASK1, apoptosis signal-regulating kinase 1; BAX, BCL2-Associated X; Bcl-2, B-cell lymphoma-2; BMP, Bone Morphogenetic Protein; BMPR-II, Bone Morphogenetic Protein Receptor Type II; Ca^2+^, intracellular free Ca^2+^ concentration; cAMP, cyclic adenosine monophosphate; CD4+; T cells, Cluster of differentiation 4 positive T cells; cGMP, cyclic guanosine monophosphate; CIHH, chronic intermittent hypobaric hypoxia; C-KIT, C-kitproto-oncogeneprotein; CRP, C-reactive protein; CXCL-1, Chemokine (C-X-C motif) ligand 1; DA, Dapagliflozin; DLL4, delta-like ligand; eIF2α, eukaryotic initiation factor 2α; EndMT, endothelial-mesenchymal transition; eNOS, endothelial nitric oxide synthase; EP, ethyl pyruvate; ERK, extracellular Signal-Regulated Kinase; ERS, Endoplasmic Reticulum Stress; ET, endothelin; FGF, fibroblast growth factor; GRP78, glucose-regulated protein78; Hes1, hairy and enhancer of split1; HMGB1, High mobility group box 1; HO1, heme oxygenase 1; HPAECs, human pulmonary arterial endothelial cells; HPASMCs, human pulmonary artery smooth muscle cells; HUVECs, human umbilical vein endothelial cells; Id1, inhibitor of differentiation 1; IL, interleukin; iNOS, nitric oxide synthase; IκB, inhibitor of NF-κB; JAG1, Jagged1; Jak, janus kinases; JNK, c-jun N-terminal kinase; LATS, large tumor suppressor; LPS, lipopolysaccharide; MAPK, mitogen-activated protein kinase; MCP−1, monocyte chemoattractant protein-1; MCT, monocrotaline; mGluR5, metabotropic glutamate receptor 5; MIF, macrophage migration inhibitory factor; miR-27b, microRNA-27b; MMP, matrix metalloproteinases; mPAP, mean pulmonary arterial pressure; MPO, Myeloperoxidase; MTEP, 3-((2-Methyl-4-thiazolyl)ethynyl)pyridine; mTOR, mammalian target of rapamycin; NF-κB, nuclear factor kappa B; NO, Nitric oxide; Notch3, Notch receptor; NOX, NADPH oxidase; NP, Nitropropionic; Nrf2, nuclear factor (erythroid-derived 2)-like 2; p38 MAPK, p38 mitogen-activated protein kinase; PAB, pulmonary artery banding; PAR, pulmonary arterial remodeling; PBA, phenylbutyric acid; PDE, pPhosphodiesterase; PDGF, platelet derived growth factor; PDGFR-β, anti PDGF receptor-β; PF, paeoniflorin; PI3K, phosphatidylinositol 3 kinase; PKA, protein Kinase A; PPARγ, peroxisome proliferator-activated receptor γ; RAGE, receptor for advanced glycation end products; ROS, reactive oxygen species; RSV, respiratory syncytial virus; RV, right ventricule; RVF, right ventricular failure; RVH, right ventricular hypertrophy; RVR, right ventricle remodeling; RVSP, right Ventricular Systolic Pressure; SGLT2, sodium glucose cotransporter 2; SMAD, drosophila mothers against decapentaplegic protein; SOCS3, suppressor of cytokine signalling 3; SP, substance P; STAT3, signal transducer and activator of transcription 3; STS, sodium tanshinone II sulfonate A; SuHx, rat Sugen rats with hypoxia; T4, 3,5,3′,5′-tetraiodothyronine; TAK, transforming growth factor-β activated kinase; TGF-β, transforming growth factor-beta; TIMP, human tissue inhibitor of metalloproteinase; TLR, Toll-like receptor; TNF, tumor necrosis factor; VA, ventricular arrhythmia; VEGF, ascular endothelial growth factor; YAP, Yes-associated protein;18β-GA, 18beta-Glycyrrhetinic acid; 3-NP, 3-nitropropionic acid.

Conversely, in mouse animal models, overexpression of IL-6 enhances PH processes. In the lung-specific IL-6 overexpression transgenic mice, the vascular damage was accompanied by the activation of VEGF, proto-oncogene transcription factor (c-MYC) and MYC-associated factor X (MAX), as well as the anti-apoptotic proteins survivin and Bcl-2, while the pro-apoptotic proteins c-Jun N-terminal kinase (JNK) and p38 kinase were downregulated. The pathological manifestations of muscularization and proliferative arteriopathy were observed in the distal arteriolar vessels of PH patients (73). Overexpressed IL-6 may promote pulmonary vascular remodeling and PH development through proliferative antiapoptotic mechanisms. Similarly, injecting mice with recombinant IL-6 resulted in PH and RV hypertrophy. The results indicated that IL-6 was associated with pulmonary vascular remodeling in which PASMC proliferated excessively (74).

In addition, other studies have elucidated additional possible mechanisms by which IL-6 was involved in the PH process in animal models. The expression of IL-6R was displayed in the remodeled pulmonary vessels of mice with enhanced IL-6 accumulation. The specific IL-6R antagonists could reverse the course of experimental PH in animal models (75). Takahiro and colleagues also identified IL-21 inducible secretion by Th17 cells and CD4+ T cells as downstream signals of the IL-6 signaling pathway in PH. The deficient in the IL-21 receptor (IL-21R) were observed to be resistant to PH and had no accumulation of M2 macrophages in the lungs of Mice. The mechanism of IL-6-induced PH was associated with the accumulation of M2 macrophages in the lungs. Furthermore, the promotion of PH by IL-21 may be related to the polarization of M2 macrophages downstream of IL-6 signaling. The IL6/IL-21 signaling axis may be a potential target for the treatment of PH (10) (**Table 1**).

## 2 Pathogenesis and pathways of IL-6 in PH

### 2.1 IL-6 and the mononuclear phagocyte system

**Figure 1:**
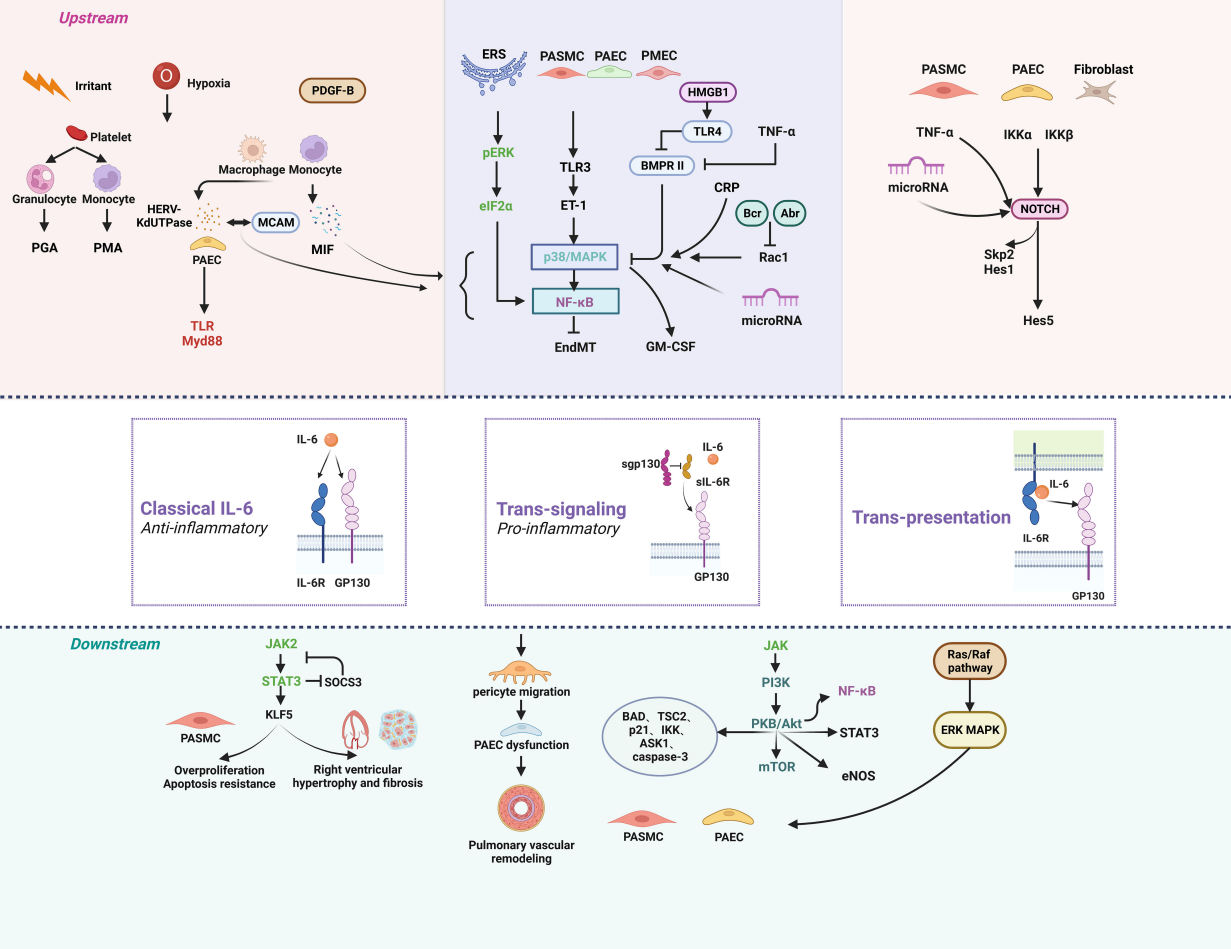
The signaling pathways of IL-6 in pulmonary hypertension. Anti-inflammatory classical signaling, pro-inflammatory trans-signaling, and trans-presentation make up the three components of the IL-6 signaling cascade. IL-6 can regulate PH through a variety of upstream and downstream pathways. *Created by bioRend.com.* Abr, active BCR-related gene; ASK1, apoptosis signal-regulating kinase 1; BAD, BCL2 associated agonist of cell death; Bcr, B-cell lymphoma; BMPR II, bone morphogenetic protein receptor type II; CRP, c-reactive protein; eIF2α, eukaryotic initiation factor 2α; EndMT, endothelial-mesenchymal transition; eNOS, endothelial nitric oxide synthase; ERK, extracellular signal-regulated kinase; ERS, endoplasmic reticulum stress; ET-1, endothelin-1; GM-CSF, granulocyte-macrophage colony stimulating factor; GP130, glucoprotein 130; HERV-K, human endogenous retrovirus K; Hes1, hairy and enhancer of split1; HES5, hes family bHLH transcription factor 5; HMGB1, high mobility group box 1; IKK, ikappaB kinase; IL-6, interleukin-6; IL-6R, interleukin-6 receptor; JAK, the janus kinases; KLF5, KLF transcription factor 5; MAPK, mitogen-activated protein kinase; MCAM, melanoma cell adhesion molecule; MIF, macrophage migration inhibitory factor; mTOR, mammalian target of rapamycin; Myd88, myeloid differentiation factor 88; NF-κb, nuclear factor kappa B; NOTCH, notch receptor; PAEC, pulmonary arterial endothelial Cell; PASMC, pulmonary artery smooth muscle cell; PDGF-B, platelet derived growth factor-B; PGA, platelet granulocyte aggregates; PI3K, phosphatidylinositol 3 kinase; PKB/Akt, protein kinase B; PMA, platelet monocyte aggregates; Rac1, Rac family small GTPase 1; Raf, Ras-associated factor; Ras, rat sarcoma virus oncogene; sgp130, soluble glucoprotein 130; sIL-6R, soluble interleukin-6 receptor; Skp2, s-phase kinase associated protein 2; SOCS3, suppressor of cytokine signalling 3; STAT3, signal transducer and activator of transcription 3; TAK1, transforming growth factor-β activated kinase 1; TLR, toll-like receptor*;* TNF-α, tumor necrosis factor-α; TSC2, TSC complex subunit 2.

The IL-6 protein has one IL-6 binding receptor protein (IL-6R) binding site and two glycoprotein 130 (GP130) binding sites (76). The major activators of IL-6 expression are IL-1β and tumor necrosis factor (TNF-α). IL-6 can be activated by three different signal transduction modes: classical signaling, trans-signaling, and trans-presentation. The classical signaling pathway contributes to the anti-inflammatory effect of IL-6, in which IL-6 binds to IL-6R and GP130 on the cell membrane to form a hexamer, which then initiates intracellular signaling. Trans-signaling contributes to the pro-inflammatory effect of IL-6, which refers to the combination of IL-6 with SIL-6R in serum or tissue fluid and GP130 present on the surface of most cells. IL-6 trans-presentation means that membrane-bound IL-6R (mIL-6R) on dendritic cells binds to IL-6 and is later presented to T cells expressing GP130, resulting in pathogenic TH17 cells (77). The hexameric complex of IL-6 with IL-6R and GP130 activates Janus kinase (JAK), which in turn activates three possible downstream signaling pathways. First, JAK induces autotyrosine phosphorylation and subsequently activates signal transducer and activator of transcription 3 (STAT3) (78). Second, JAK activates the Ras/Raf pathway, which subsequently leads to hyperphosphorylation of MAPK and increases its serine/threonine kinase activity (78). Third, JAK activates phosphatidylinositol-3-kinase (PI3K) and nuclear factor kappa-B (NF-κB) *via* the PI3K/AKT pathway (**Figure 1**) (79).

In human immunity, the main functions of IL-6 are to stimulate the activation and proliferation of B cells, stimulate T-cell proliferation and cytotoxic lymphocyte activation, stimulate hepatocytes to synthesize acute phase proteins to participate in inflammatory responses, and promote blood cell development (80, 81). Therefore, IL-6 is affected in severe burns, tumors, hepatitis, and diseases of the nervous system, immune system, blood system, kidney system, and vascular system (82–85). Macrophages and pulmonary arterial smooth muscle cells (PASMCs) are regulated by the classical IL-6 signaling pathway (86). In young male SV129 mice, increased IL-6 in plexogenic lesions may be associated with increased recruitment of macrophages (87). Macrophage M1 polarization mainly secretes proinflammatory factors in the early stage of inflammation. Macrophage M2 polarization mainly expresses anti-inflammatory factors, inhibits inflammatory factors and anti-inflammatory factors in the later stage of inflammation, and carries out tissue repair and reconstruction. IL-21 is the downstream target of IL-6 signaling, which can promote the polarization of PH alveolar macrophages toward M2 phenotype to excrete matrix proteins, and exert anti-inflammatory, angiogenic and tissue repair effects by stimulating the proliferation of human PASMCs (HPASMCs) (10). Studies have found that mononuclear cell derived macrophages in male PH mice and male PAH patients have a decreased M1/M2 ratio (**Figure 1**) (88).

Hypoxia, as the main driver of PH, lead to increased levels of macrophage migration inhibitory factor (MIF) in the monocyte-macrophage system, thereby activating NF-κB and p38 mitogen-activated protein kinase (p38MAPK) pathways to regulate IL-6 (37, 89, 90). Thyroxine T4 is a natural ligand inhibitor of MIF, which can inhibit the inflammatory activity of MIF by blocking the MIF site, and different anti-MIF treatments achieve good efficacy in animal models (**Table 1**) (37). Circulating monocytes and macrophages in the adventitial of the pulmonary arteries of PH patients express high levels of human endogenous retrovirus K deoxyuridine 5’-triphosphate pyrophosphatase (HERV-K dUTPase) and activate the Toll-like receptor 4 (TLR4) myeloid differentiation primary response-88 signaling pathway in pulmonary artery endothelial cells (PAECs) after vesicle release, leading to IL-6 elevation (**Figure 1**) (91). HERV-K dUTPase could also interact with melanoma cell adhesion molecule (MCAM) followed by NF-κB and p38/MAPK activation and promote IL-6 release (91). The viral Toll-like receptor 3 (TLR3) expressed in HPASMCs could release endothelin-1 (ET-1) in large quantities after activation, which leads to an increase in Ca^2+^ in monocytes and the release of IL-6 through p38/MAPK pathway (92–94). Peripheral blood platelets in patients with WHO Group 1 and 4 PH are activated by aggregation with monocytes and granulocytes to form platelet-monocyte aggregates (PMAs) and platelet-granulocyte aggregates (PGAs), thereby promoting IL-6 production and release, but the mechanism remains unclear (95). Human Regnase-1 is a ribonuclease that downregulates IL-6 by degrading IL-6 mRNA (96). Regnase-1 is expressed less in PH subtype, while mice lacking Regnase-1 in alveolar macrophages develop severe PH, and transcriptomic analysis has shown that IL-6 is a potential target for Regnase-1 in alveolar macrophages of PAH patients, demonstrating that Regnase-1 might be another mediator of IL-6 in monocytes/macrophages in PH (**Figure 1**) (97).

### 2.2 IL-6 and JAK/STAT signaling pathways

JAK, also known as Janus Kinase, is a non-receptor tyrosine protein kinase. It can mediate signal molecules activation by IL-6. STAT is recruited and activated so that it enters the nucleus in the form of a dimer to bind to target genes, regulate downstream gene transcription, and participate in many important biological processes, such as cell growth, differentiation, apoptosis, and immune regulation. The activated JAK protein phosphorylates the receptor and itself, and the phosphorylation site binds to the STAT and adaptor proteins that link the receptor to MAPK, PI3K/AKT, and other pathways (**Figure 1**) (98).

JAK2 is overactivated and expressed abundantly in HPASMCs from IPAH patients (34). IL-6, IL-6R and IL-6R subunit β (GP130) form a hexamer, and activation of the CD52/STAT3 signaling pathway is a classic IL-6 signaling pathway that occurs in macrophages and PASMCs (86). STAT3 signaling can inhibit the transcription of cytokine signal suppressor 3 (SOCS3), which is involved in IL-6 signaling (72). IL-6 also activates Kruppel-like factor 5 (KLF5) in HPASMCs through STAT3 signaling, promotes the upregulation of cyclin B1 and the proliferation of PASMCs, triggers the expression of hyperpolarized mitochondrial membrane potential of survival proteins, and reduces PASMC apoptosis (99). In Schistosoma-induced PAH, IL-6 can induce upregulation of the IL-6-STAT3-nuclear factor of activated T cells c2 (NFATc2) pathway, which has a protective effect on intimal remodeling (100). However, it is generally believed that IL-6 ultimately induces the transcription of proinflammatory and proangiogenic genes through STAT signaling and promotes PH progression. Moreover, IL-6 can induce RV hypertrophy and fibrosis in PH rats through the JAK2/STAT3 signaling pathway (101). Finally, miR-125a-5p ameliorates MCT-induced PH by targeting the TGF-β1 and IL-6/STAT3 signaling pathways (**Figure 1**) (102).

The Jak1 and Jak2 inhibitor ruxolitinib can effectively reduce the proliferation and migration of HPASMCs induced by IL-6, and reduce pulmonary vascular remodeling (34). Prostaglandins could not only stimulate intracellular 3’,5’-cyclic adenosine monophosphate (cAMP) levels to induce vasodilation, but also induce SOCS3, thus inhibiting IL-6-induced inflammation and vascular remodeling, which is also considered as one of the current therapeutic ideas for PH (72) (**Table 1**).

### 2.3 IL-6 and NF-κB signaling pathways

NF-κB is a nuclear transcription factor that plays a key role in the cellular inflammatory response and immune response (103). In the contexts of aging, obesity, stress, infections, injuries and smoking, activation of NF-κB and STAT3 in nonimmune cells triggers a positive feedback loop of the IL-6-STAT3 axis to NF-κB, which is called the IL-6 amplifier (104). IL-6 also inhibits NF-κB levels through the downstream PI3K/AKT signaling pathway (**Figure 1**) (105).

TLR4, one of the TLR family, activates downstream NF-κB and/or MAPK signaling pathways, and induces the production and secretion of IL-6 after lipopolysaccharide (LPS) challenge (47). Knocking out the E26 transformation-specific 2 gene can inhibit MAPK/NF-κB signals and negatively regulate IL-6 in inflammation (106). TLR4/NF-κB expression levels can even reflect the severity of PH in COPD patients (107). BMPR II deficiency confers resistance to growth inhibition by TGF-β in PASMC (108). Wynants et al. found NF-κB pathway is involved in CRP-induced effects on PASMCs in chronic thromboembolic pulmonary hypertension (CTEPH). The NF-κB pathway inhibitor pyrrolidinedithiocarbamate ammonium (PDTC) can reduce IL-6 secretion (109).

The endoplasmic reticulum (ER) is a central organelle of the mammalian intracellular membrane system and is extremely sensitive to stress that affects intracellular energy levels, oxidative states, or abnormal calcium concentrations. When cells are subjected to certain stresses (such as infection, hypoxia, drug toxicity, etc.), ER dysfunction causes the accumulation of unfolded proteins or misfolded proteins in the lumen of the endoplasmic reticulum and a state of calcium imbalance: that is, ER stress (ERS). Early ERS exerts a protective effect and is beneficial to RV function; if this imbalance exceeds the body’s ability to regulate itself, it will eventually lead to apoptosis of cardiomyocytes and RV dysfunction (48). ERS activates and induces IL-6 elevation through the ERK/eIF2α/NF-κB signaling pathway, promotes pulmonary vascular remodeling, and participates in PH development (38). Increased pericyte coverage mediated by endothelial-derived fibroblast growth factor-2 and IL-6 is a source of smooth muscle-like cells in PH (110). Hypoxia leads to upregulation of miR-27b expression in HPAECs, inhibits peroxisome proliferator-activated receptor gamma (PPARγ), induces IL-6 secretion through the NF-κB signaling pathway, and aggravates HPAEC dysfunction (35). The low expression of miRNA-340-5p in the plasma of patients with PH led to the activation of the NF-κB pathway and induced the upregulation of IL-6, thereby promoting the inflammatory response, proliferation and migration of PASMCs and participating in PH progression (**Figure 1**) (111).

The treatment of PH through the NF-κB signaling pathway is a hot spot in current research. The traditional Chinese medicine SroloBzhtang (SBT) produces anti-inflammatory effects by inhibiting the MAPK/NF-κB signaling pathway, resulting in a decrease in IL-6 (47). Dapagliflozin (DA) can significantly reduce Toll-like receptor 4 (TLR4) and NF-κB levels in monocrotaline-induced PH rats (MCT-PH) and alleviate PH-related symptoms (49). By attenuating NF-κB activation, 10 μmol/L atorvastatin completely eliminated the increase in CRP-induced IL-6 in cultured HPASMCs, proving that atorvastatin and similar drugs may be able to control the PH inflammatory response by inhibiting NF-κB activation (71). 18β-glycyrrhetinic acid (18β-GA) significantly reduces the accumulation of misfolded proteins in rat lung tissue, inhibits ERS activation, inhibits NF-κB migration to the nucleus, and increases the expression of the inhibitor of NF-κB (IκB), resisting vascular remodeling and alleviating PH (38). 4-Phenylbutyric acid (PBA) can inhibit ER stress and alleviate RV remodeling and dysfunction (48). Sevoflurane and thymoquinone can reduce IL-6 and TNF-α by inhibiting the NF-κB and MAPK pathways, partially inhibiting pulmonary vascular remodeling and right ventricular hypertrophy in PH rats (51, 52). Hypoxia leads to upregulation of miR-27b in HPAECs, inhibits the expression of PPARγ and induces the secretion of IL-6, while fibroblast growth factor 21 (FGF21) can reduce the expression of IL-6 by inhibiting miR-27b, thus alleviating PH and right ventricular hypertrophy (RVH) (35, 36). Chronic intermittent hypobaric hypoxia attenuates MCT-PH by modulating TNF-α and IL-6 and suppressing the NF-κB/p38 pathway (50) (**Table 1**).

### 2.4 IL-6 and MAPK signaling pathways

Mitogen-activated protein kinases (MAPKs) are a group of serine-threonine kinases that are important transducers of signals from the surface of eukaryotic cells to the interior of the nucleus, including ERK, p38, and JNK (112). Activation of MAPK signaling can drive the remodeling and inflammation of the pulmonary vasculature and resist apoptosis of PASMCs (**Figure 1**).

In PH, the current research mainly involves the p38 subfamily. Hypoxia leads to increased MIF levels in fibroblasts, endothelial cells and mononuclear macrophages, which can induce the activation of the p38/MAPK pathway (91). Hypoxia itself activates p38, JNK, and ERK in the pulmonary arteries (113). Activated p38/MAPK can phosphorylate inhibitors of NF-κB, thereby migrating activated NF-κB into the cell nucleus and regulating IL-6 expression at the transcriptional and posttranscriptional levels. The expression of p38/MAPK and IL-6 in the pulmonary vessels of IPAH patients increased, as demonstrated the fact that p38/MAPK antagonists was able to reduce IL-6 in PH rat models (114). Upregulation of IL-6 in both transgenic mice and HPASMCs cultured with siRNA against BMPR II could be abolished with p38 (MAPK) inhibitors, highlighted the interaction of IL-6 and the BMP pathway in PASMCs (16). BMPR II silencing resulted in impaired endothelial barrier function and activation of p38MAPK in human pulmonary microvascular endothelial cells (HPMECs) (115, 116). Inhibition of MCT-induced BMPR II downregulation and phosphorylation of the transforming growth factor-β (TGF-β)-activated kinase 1 (TAK1)-MAPK/NF-κB pathway prevents the proliferation and migration of HPASMCs and reverses endothelial-to-mesenchymal transition (EndMT), which can be attenuated by paeoniflorin (39). Downregulated BMPR-II induces the translation of granulocyte macrophage colony-stimulating factor (GM-CSF) mRNA *via* p38/MAPK activation, increasing the recruitment of perivascular inflammatory cells and intensifying PH (117). The activity of p38MAPK signaling is tightly regulated by the inactivation of dual-specificity phosphatase 1 (DUSP1) (**Figure 1**). Phosphorylated/activated MAPKs, including p38MAPK, induce the expression of DUSP1. DUSP1 can dephosphorylate JNK, p38 and ERK. However, the impaired p38/MAPK/DUSP1 pathway in PH causes unregulated activation of p38/MAPK, resulting in an IL-6 surge and HPASMC proliferation and migration (118). DUSP1 expression can be increased by prostacyclin receptor agonists—prostacyclin and MRE-269 (an active metabolite of selexipag).

Bcr and Abr are GTPase activating proteins that specifically downregulate the activity of the small GTPase Rac in restricted cell types *in vivo*. Rac1 is expressed in PASMCs and involved in the pathogenesis of PH. Bcr and Abr can reduce p38/MAPK phosphorylation, IL-6 production, HPASMC proliferation, and leukocyte oxidative stress by specifically downregulating Rac1 activity in HPASMCs (119). The downregulation of miRNA-126 affects MAPK activation, reduces microvascular density through the vascular endothelial growth factor (VEGF) pathway, and promotes the decompensated RV (**Figure 1**) (120).

There are many more drugs involved in the MAPK pathway. Thymoquinone, Sevoflurane, and Baicalein all downregulate IL-6 levels by inhibiting the p38/MAPK and NF-κB pathways, with the traditional Tibetan medicinal formula SroloBzhtang (SBT) (47, 51–53). Capsaicin pretreatment reversed PH by alleviating inflammation *via* p38MAPK pathway (54). Aspirin weakens PH by inhibiting the ERK signaling pathway (55). Oral administration of the apoptosis signal-regulating kinase 1 (ASK1) inhibitor GS-444217 reduced phosphorylation of p38 and JNK in rat cardiomyocytes, reduced the remodeling of the pulmonary vasculature and RV, and prevented PH progression (33). Resveratrol (trans-3, 5, 4’-trihydroxystilbene) can inhibit IL-6 expression and PH vascular remodeling by inhibiting multiple signaling pathways, such as ERK/MAPK, NF-κB, bone morphogenetic protein (BMP), PI3K/AKT, and hypoxia inducible factor-1α (HIF-1α) (32). The metabotropic glutamate receptor 5 (mGluR5) inhibitor 3-((2-methyl-4-thiazolyl) ethynyl) pyridine (MTEP) inhibits the signaling cascade involving PI3K/AKT, p38/MAPK, angiopoietin 2 and VEGF (56). Baicalin reduces chronic hypoxia-induced PH by downregulating the p38/MAPK/MMP-9 pathway (121). Low-dose masitinib resisted vascular remodeling in lung tissue by blocking MAPK pathway (**Table 1**) (57).

### 2.5 IL-6 and PI3K/AKT signaling pathways

Phosphatidylinositol 3-kinases (PI3K) are intracellular phosphoinositide kinases with phosphatidylinositol kinase activity and serine/threonine (Ser/Thr) kinase activity that consist of a dimer that regulates subunit p85 and catalyzes subunit p110. The p110α subunit is a pathogenic signaling pathway downstream of multiple receptor tyrosine kinases (RTKs) and a key regulator of HPASMC proliferation, migration, and survival. Pharmacological inhibition of p110α can inhibit vascular proliferation, induce apoptosis, and reverse vascular remodeling and PH development (122). RTKs and other cytokines activate PI3K, and cause downstream PKB/AKT activation. Downstream substrates of the PI3K/AKT pathway include the BCL-2 family members BAD, tuberin, p21, IκB kinase (IKK), and ASK1. The effects of the PI3K/AKT signaling pathway mostly cause cell proliferation and apoptosis resistance. The hexameric complex of IL-6 with IL-6R and GP130 activates JAK, which in turn activates the PI3K/AKT signaling pathway. In MCT-PH rat, IL-6 and PI3K/AKT signaling pathways increase with PH progression, and IL-6 can serve as a marker for the PH chronic inflammatory (123). Hypoxia caused increase of IL-6 in rat lung tissue, activated PKB/AKT/mTOR pathway to inhibit autophagy, and thus leading to the proliferation, migration of HPASMCs and HPAECs (62, 124). AKT induces phosphorylation of the tuberin to activate the mTOR pathway, which itself can be activated by hypoxia. At the same time, PDGF-B, which is elevated in patients with PAH, can activate STAT3 through the PKB/AKT signaling pathway, inhibit apoptosis, and promote disease progression (125). IL-6-activated JAK can also activate STAT3. Tripartite motif 32 (TRIM32), a member of the TRIM family, is involved in cardiovascular diseases (126). TRIM32 protein inhibits cardiomyocyte hypertrophy by acting as a key regulator of cell viability and apoptosis in cardiomyocytes (126). TRIM32 levels in plasma of patients with PH were lower than that of control subjects. Overexpression of TRIM32 enhanced the apoptosis of hypoxia-induced PASMCs through the PI3K/AKT signaling pathway (127). PI3K/AKT can induce eNOS activation, exacerbating PH by protein kinase G nitration (**Figure 1**) (128).

Atorvastatin alleviates the symptoms of PH in rats by activating the PI3K/AKT signaling pathway by effectively upregulating the expression of apoptosis proteins (63). Sodium tanshinone II sulfonate A (STS) alleviates hypoxia-induced PH by promoting apoptosis, inhibiting the PI3K/AKT/mTOR pathway, upregulating autophagy, and inhibiting inflammatory responses (62). Resveratrol and 3-((2-methyl-4-thiazolyl)ethynyl)pyridine inhibit multiple signaling pathways, including the PI3K/AKT pathway, to treat PH (32, 56). Luteolin inhibits PI3K/AKT by downregulating the expression of LATS1, YAP and PDGF-B and is therapeutic for experimental PH (40). Telmisartan, genistein, and tanshinone IIA all inhibit PH development by the PI3K/AKT/eNOS pathway (**Table 1**) (43, 58, 59).

### 2.6 IL-6 and BMP signaling pathways

**Figure 2:**
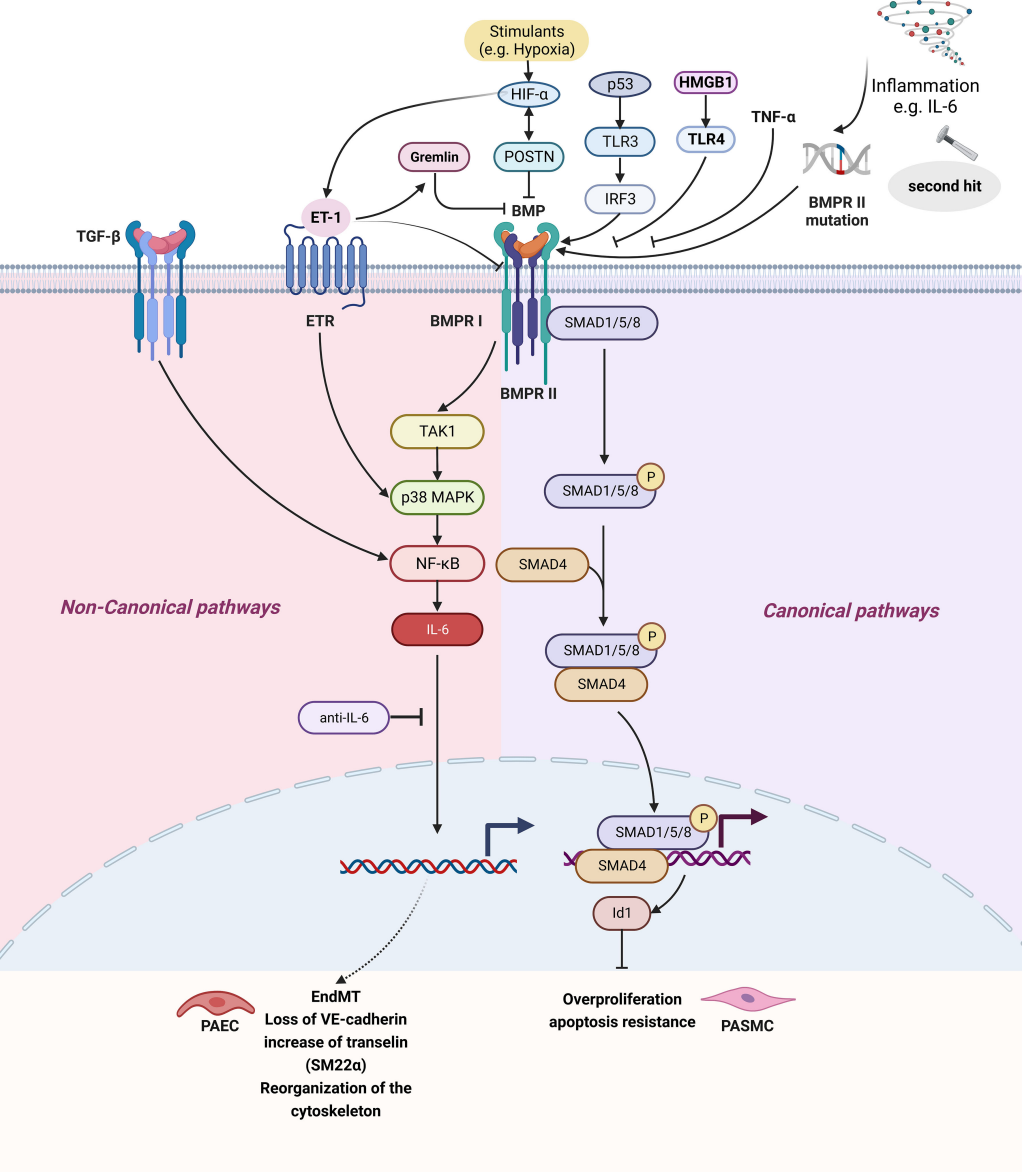
The IL-6 and BMP signaling pathway in pulmonary hypertension. BMP signaling pathways can be divided into signaling pathways and non-canonical signaling pathways. The classical pathway refers to BMP II forming complex with BMPR II, BMPR II promoting BMPR-I activation, three forming polymer, BMPR I causing Smad1/5/8 phosphorylation in the cytoplasm and binding Smad4 forming complex, finally entering the nucleus regulating target gene, affecting cell proliferation, Inhibition of BMPR II can lead to inhibition of the BMP classical pathway. Non-classical pathways are BMPR I activation TAK1/MAPK signaling pathways and activation of NF- κB, causing IL-6 secretion. *Created by bioRend.com.* BMP, bone morphogenetic protein; BMPR, bone morphogenetic protein receptor; EndMT, endothelial-mesenchymal transition; ET-1, endothelin-1; ETR, endothelin receptor; HIF, hypoxia-inducible factor; Id1, inhibitor of differentiation 1; IL-6, interleukin-6; IRF3, interferon regulatory factor 3; MAPK, mitogen-activated protein kinase; NF-κb, nuclear Factor kappa B; P, phosphorylation; p53, tumor protein 53; PAEC, pulmonary arterial endothelial cell; PASMC, pulmonary artery smooth muscle cell; POSTN, periostin; SARA, smad anchor for receptor activation; SM22α, smooth muscle 22 alpha; SMAD, SMAD family member; Smurf2, SMAD specific E3 ubiquitin protein ligase 2; TAK1, transforming growth factor-β activated kinase 1; TNF-α, tumor necrosis factor-α; TGF-β, tumor necrosis factor-β; TLR, toll-like receptor; HMGB1, high mobility group box 1; VE-cadherin, vascular endothelial cadherin.

According to structural and functional similarities, the TGF-β superfamily is divided into TGF-β and BMP subfamilies. It has been recognized that BMP pathway is related to IL-6 elevation and disease progression in PH patients. BMP usually combines with BMPR II, and activates downstream classical or non-canonical signaling pathways. The canonical pathway involves phosphorylated Smad1/5/8 binding to Smad4 in the cytoplasm to form a complex and enter the nucleus, which affects cell proliferation, migration, and apoptosis by regulating target genes (129). In the non-canonical signaling pathway, BMP activates the signaling pathway of TAK1, a member of the MAP3K family, through BMPR I and activates NF-κB and JAK pathways (**Figures 1** and **2**) (130, 131).

Currently, the roles of BMPR II in PH research have been well studied. It is known that only 20-30% of people with BMPR II mutations exhibit hereditary PH (46). BMPR II silencing has been proven to enhance the activation of TNF-α on p38MAPK and endothelial dysfunction injury (115). POSTN (Periostin) is an extracellular matrix protein involved in tissue remodeling in response to injury and a contributing factor in tumorigenesis and downregulation of BMPR II by activation of HIF-1α, which was induced by overexpression of POSTN with a positive feedback loop, along with the increased production of ET-1 and VEGF in HPAECs and excessive proliferation and migration of HPASMCs and HPAECs (**Figure 2**) (132). In the pathological state, ET-1 affects classical BMP signaling by activating p38/MAPK, downregulating BMPR II and expressing gremlin (116). Increased expression of gremlin1, a secreted glycoprotein antagonizing BMPR II signaling through the binding of BMPs, was recently reported to contribute to the development of experimental murine hypoxic PH (116). BMPs can upregulate the expression of potassium two pore domain channel subfamily K member 3 (KCNK3) *via* the classical BMP-BMPR II-Smad1/5pathway, and the dysfunction and/or downregulation of BMPR II and KCNK3 observed in PH work together to induce aberrant changes in the PASMC phenotype, providing insights into the complex molecular pathogenesis of PH (133). PAEC-specific p53 knockout exaggerated PH, and clonal expansion reduced p53 and TLR3 expression in rat lung CD117+ ECs. Reduced p53 degradation (Nutlin 3a) abolished clonal PAEC expansion, induced TLR3 and BMPR II, and ameliorated PH. Polyinosinic/polycytidylic acid increased BMPR II signaling in PAECs *via* enhanced binding of interferon regulatory factor-3 (IRF3) to the BMPR II promoter and reduced PH in p53^-/-^ mice, but not in mice with impaired TLR3 downstream signaling. The p53/TLR3/IRF3 axes can be used to regulate the expression and signaling of BMPR II in HPAECs (134).

BMPs are involved in both classical and non-classical pathways. The BMPR II mutation inhibits the classical pathway of BMPR II-assisted BMPR I activation, thereby increasing the proportion of non-classical pathways. The non-classical pathway induces TGF-β1 secretion, IL-6 expression is increased through NF-κB signaling, and IL-6 inhibits the antiproliferative function of TGF-β1 (**Figure 2**) (108). The IL-6 and BMP pathways are mainly non-classical signaling pathways. In PH patients with BMPR II mutations, HPASMCs produced more IL-6 after stimulation by LPS, demonstrating that BMPR II was associated with elevated IL-6 in patients with PH (46). HPAH PASMCs exhibited enhanced IL-6 and IL-8 induction by TGF-β1, an effect reversed by NF-κB inhibition (108). This effect is Smad-independent but is associated with inappropriately altered NF-κB signaling and enhanced induction of IL-6 and IL-8 expression. Anti-interleukin therapies may neutralize this inappropriate response and restore the antiproliferative response to TGF-β1 (108). BMPR II mutation carriers develop PH, suggesting that the most important function of BMPR II mutation is to cause susceptibility to a “second hit”, such as dysregulated inflammation, particularly by the IL-6 (16). In both transgenic mice and PASMCs cultured with siRNA against BMPR II, the BMP pathway regulates IL-6 in pulmonary tissues, and conversely, IL-6 regulates the BMP pathway. A complete negative feedback loop between IL-6 and BMP suggested that an important consequence of BMPR II mutations may be poor regulation of cytokines, and thus vulnerability to an inflammatory second hit (16). Additionally, in PAECs, prolonged EndMT signaling characterized by a loss of VE-cadherin, induction of transgelin (SM22α), and reorganization of the cytoskeleton were found, accompanied by sustained elevation of proinflammatory, prohypoxic, and proapoptotic signaling. Among them, IL6-dependent signaling was identified to be the central mediator required for the BMP9-induced phenotypic change in PH PAECs. BMP9-induced EndMT by an IL6 capturing antibody prevented mesenchymal transformation and maintained a functional PAEC phenotype in PH PAECs (**Figure 2**) (16, 135).

Dexamethasone, which is commonly found in clinical practice, can prevent and reverse experimental PH by restoring BMPR II expression and reducing IL-6 levels, inhibiting the proliferation of HPASMCs, and improving blood flow and pulmonary vascular remodeling (60). The HMGB1 inhibitors saquinavir and glycyrrhizic acid and the TLR4 inhibitor TAK-242 can restore BMPR II signaling and alleviate PH development in rats. This is because HMGB1 promotes PASMC proliferation, migration and pulmonary vascular remodeling through activation of the ERK1/2/Drp1/autophagy/BMPR II/Id1 axis (45, 136). BMPR II deficiency promotes an exacerbation of inflammatory responses *in vitro* and *in vivo*, while chronic administration of superoxide dismutase mimetic (TEMPOL) alleviates inflammation and PH (46). Both baicalin and isorhamnetin can improve the hemodynamics by improving BMPR II expression in experimental rats, and inhibit HPASMCs proliferation induced by IL-6 and TNF-α through the BMP pathway (41, 42). Paeoniflorin (PF) can prevent the proliferation and migration of HPASMCs by inhibiting BMPR II downregulation and TAK1 phosphorylation, and block EndMT by reversing inflammatory factors in HPAECs (**Table 1**) (61, 86).

### 2.7 IL-6 and notch signaling pathways

The Notch signaling pathway is composed of Notch receptor, Notch ligand (DSL protein), intracellular effector molecule (CCL-DNA binding protein) and other regulatory molecules. It is an important pathway for communication between adjacent cells and regulation of cell development. Notch receptors include Notch1/2/3/4, while Notch ligands include Delta-like1/3/4, Jagged1/2, and CSL-DNA-binding proteins [CBF-1/Suppressor of hairless (Su (H))/Lag] (137, 138). The Notch signaling pathway eventually forms the NICD/CSL transcriptional activation complex, which activates target genes of the hairy and enhancer of split (HES), HEY, HERP and other transcriptional inhibitory factor families and plays a biological role (138, 139).

Currently, there is substantial evidence that the Notch signaling pathway regulates IL-6 levels in Graves’ ophthalmopathy, fibroblasts, endometriotic lesions and breast tumor cells of pancreatic cancer (140–143).

The indispensable involvement of Notch1 in the arterial endothelial phenotype and angiogenesis provides intriguing prospects for its involvement in the pathogenesis of PH. The Notch ligand Delta-like 4 (DLL4), which is least expressed in HPASMCs in patients with PH, inhibits Notch3 cleavage and signaling and delays vascular smooth muscle cell proliferation (144). Delta-like 4 neutralizing antibodies (DLL4nAbs) can lead to impaired HPAEC barrier function and inhibit Notch1 activation, so PH occurs in 14 to 18% of patients treated with DLL4nAbs (145). Increasing the level of DLL4 in patients can help delay disease progression through two pathways: Notch1 and Notch3. This partly supports the fact that the primary focus of the Notch signaling pathway in lung disease is Notch1 and Notch3 (146). Hypoxia can also induce increased expression of Notch1 in HPAECs, while Notch1 promotes HPAEC proliferation by downregulating p21 and inhibits apoptosis through Bcl-2 and Survivin (147). Upregulated miR-18a-5p in patients with PAH promotes the proliferation and migration of HPASMCs by inhibiting Notch2 expression (148). Notch2 reduces the activation of Notch1 through retinoblastoma gene-transcription factor E2F-1 (Rb-E2F-1)-mediated signaling and induces the proliferation and antiapoptotic resistance of HPAECs (149). On one hand, TNF-α selectively reduces BMPR II transcription and mediates posttranslational BMPR II cleavage *via* the ADAM10 and ADAM17 in PASMCs. On the other hand, TNF-α-mediated suppression of BMPR II subverts BMP signaling, leading to BMP6-mediated PASMC proliferation *via* preferential activation of the ALK2/ACTR-IIA signaling axis. Furthermore, TNF-α, *via* proto-oncogene protein tyrosine kinase (SRC) family kinases, increases pro-proliferative Notch2 signaling in HPAH PASMCs with reduced BMPR II expression. Anti-TNF-α immunotherapy reverses PH progression, restoring normal BMP/NOTCH signaling (150). Notch3 is expressed only in vascular smooth muscle cells and is involved in pulmonary vascular remodeling. Notch3-mutant cells in pediatric PAH patients have increased proliferation and viability than wild-type cells, thereby affecting the Notch3/HES5 signaling pathway to induce the pathogenesis of PH (151). Notch3 signaling also stimulates cell proliferation in pulmonary vascular cells *via* S-phase kinase associated protein 2 (Skp2) and Hes1 (152, 153). Activation of the Notch3 signal also enhances transient receptor potential canonical 6 (TRPC6), allowing Ca^2+^ to enter HPASMCs through store-operated Ca^2+^ channels, combined with Ca^2+^ sensing receptor activation-mediated TRPC6 receptor-operated Ca^2+^ channels, which cause persistent vasoconstriction of the pulmonary arteries (154, 155). Overexpression of Jagged1 and chemokine C-C motif ligand 2 (CCL2) in HPASMCs of PAH induces Notch3 signaling and leads to cell proliferation and PH development (**Figure 1**) (152, 156).

In recent years, more studies have confirmed the role of miRNAs in regulating NOTCH in PH. For example, miR-27b upregulation was found in PH patients associated with congenital heart disease (157). MiR-27b upregulation can inhibit PPAR-γ expression, leading to endothelial dysfunction and remodeling (158). However, *in vitro* studies have shown that overexpression of miR-27b reduces the expression of Notch1 protein, which can delay the development of PH (157). Although the study did not involve Notch1 protein expression in patients, it is speculated that cell density-dependent activation is the main activation mode of Notch1 *in vivo*. MiR-30d-5p was downregulated in both patients with PAH and animal models compared with control groups. The regulatory mechanism underlying PASMCs may be *via* the Notch 3 signaling pathway (159).

Evidence that NOTCH and IL-6 are activated in mouse embryonic fibroblasts NIH 3T3 cells in a density-dependent manner has been reported. Notch signaling regulates cell density-dependent apoptosis of NIH 3T3 cells through an IL-6/STAT3-dependent mechanism (160).

Although there is no direct evidence related to how the Notch signaling pathway regulates IL-6 levels in patients with PH, it is deduced that Notch signaling may also exert an effect on IL-6 expression in PH. For example, enhanced Notch signaling mediates IL-6 levels through noncanonical Notch signaling, which is regulated by inhibitors of NF-κB kinase subunits α and β (IKKα and IKKβ) in the p53 and NF-κB signaling pathways but does not participate in the NF-κB signaling pathway (140). As mentioned earlier, the NF-κB signaling pathway mediates IL-6 to play an important role in PH. Therefore, Notch signaling may have an effect on IL-6 expression in PH. In pancreatic cancer, IL-6 induces the expression of Jagged-1/2 through Jak/STAT signaling, which enhances the expression of IL-6 mRNA in fibroblast cell lines through the NF-κB pathway to form a positive feedback loop and stimulates the production of platelets to form a hypercoagulable state (142). The hypercoagulable state is one of the most important factors affecting the PH development.

Atorvastatin effectively inhibits the expression of Notch1 and IL-6 (64). Hesperidin may reduce respiratory syncytial virus (RSV)-induced lung inflammatory damage in mice with bronchiolitis by inhibiting the Jagged1/Notch1 signaling pathway and promoting M2-type polarization of macrophages (70). Asiatic acid mitigates LPS-induced damage by inhibiting the activation of the Notch signaling pathway (68). Sildenafil inhibits RVH and pulmonary vascular remodeling by inhibiting Notch3 signaling (**Table 1**) (65).

### 2.8 IL-6 and HMGB1

High mobility group box 1 (HMGB1) is a highly conserved nuclear protein widely distributed in mammalian cells with advanced proinflammatory effects. HMGB1 is significantly upregulated in the pulmonary arteries of patients with IPAH (45). Serum HMGB1 levels were significantly increased in hypoxia-induced neonatal PH patients, and the changes in HMGB1 were positively correlated with serum TNF-α and IL-6 levels (161). Both hypoxia and HMGB1 can promote IL-6 expression, and HMGB1 can promote IL-6 expression in HPASMCs and HPAECs (162). In Huntington’s disease studies, the HMGB1/TLR4/NF-κB signaling pathway has been shown to be key to the regulation of IL-6 levels (69). Meanwhile, HMGB1 can promote advanced glycation end products (RAGE) expression, suppress BMPR II signals through the HMGB1/TLR4 channel, and promote PH development (45, 162).

The HMGB1 inhibitors saquinavir and glycyrrhizic acid and the TLR4 inhibitor TAK-242 can shut down the HMGB1/TLR4/NF-κB signaling pathway and affect the proliferation and migration of HPASMCs caused by HMGB1 signaling (**Figures 1** and **2**) (45). Baicalin administration significantly attenuated mPAP and RV hypertrophy in infant rats with PH. Expression levels of HMGB1, RAGE, IL-6 and TGF-β1 in lung tissue were dramatically decreased by baicalin in a dosage-dependent manner. Activation of PPARγ that inhibited HMGB1/RAGE inflammatory signaling was involved (66). Ethyl pyruvate (EP) also affects the proliferation of HPASMCs by inhibiting HMGB1/RAGE expression and alleviating PH (67). Interestingly, HMGB1 is released by either HPAECs or HPASMCs that underwent necrotic cell death, although only HPASMCs produce HMGB1 during apoptosis. Moreover, only HPASMC death induced a release of dimeric HMGB1, found to be mitochondrial reactive oxygen species dependent, and TLR4 activation (**Table 1**) (163).

### 2.9 Iron deficiency

Patients with IPAH are more likely to develop unexplained iron deficiency, and IL-6 levels are associated with iron levels and transferrin saturation (164). IL-6 causes hepcidin transcription and release, inhibits iron transporter-mediated iron excretion in iron cells, contributes to the proliferation of HPASMCs, and regulates the hemoglobin receptor CD163 on HPASMCs, affecting iron uptake by hemoglobin to HPASMCs, and thus leading to cell proliferation (**Table 1**) (165).

## 3 Discussion

IL-6 plays a huge role in the occurrence and development of PH. In PH patients, IL-6 is not only an inflammatory factor, but also serves dual proinflammatory and anti-inflammatory roles and is also the “mediator” that leads to the onset and progression of PH. IL-6 transmits information by participating in various signaling pathways, inducing immune reactions in the body and causing pathological damage. By modulating the IL-6 signaling pathway, cell proliferation, migration and pulmonary artery remodeling in PH can be improved or even reversed, improving the quality of life of patients and reducing mortality.

Simpson and Hirsch have validated the association between IL-6 and connective tissue diseases associated with PAH (21, 26). IL-6 levels can be used to identify all stages and severity of scleroderma (SSc). The gene expression and cytokine profiles of limited SSc-PAH patients suggest the presence of activated monocytes and show markers of vascular injury and inflammation. Among them, IL-6 could serve as a biomarker of PAH involvement in limited SSc. Compared with SSc patients with a short course or no complications from SSc, IL-6 levels were higher in patients with SSc with PAH ≥ 5 years or with severe complications (166, 167). In the early stages of SSc, IL-6 leads to damage-associated molecular patterns that maintain inflammation and play an important role in fibroblast transformation development, and the use of tocilizumab can inhibit IL-6 by binding to IL-6R, thus inhibiting inflammation and fibrosis (168). However, IL-6 levels did not predict the response of PH to tocilizumab, and PVR did not change before and after IL-6 signaling block treatment (169). It is speculated that tocilizumab combined with IL-6R inhibited classical anti-inflammatory IL-6 signaling and affected proinflammatory IL-6 trans-signaling, resulting in the maintenance of pulmonary vascular tone.

Japanese researchers found that increased plasma IL-6 levels in patients with CTEPH were positively correlated with endotoxin levels, and the intestinal flora of patients was different from that of healthy people. Endotoxin causes increased activity of IL-6 and acid sphingomyelinase (aSMase) in HPASMCs, promotes pulmonary vasoconstriction, induces endothelial dysfunction and enhances the contractile response to serotonin, ultimately leading to PH in patients with acute respiratory distress syndrome (ARDS) (170). It has also been shown that both endotoxin and IL-6 can increase PVR, causing cirrhosis leading to PH (171). In other words, the reasons for the increase in IL-6 in PH patients are diverse. IL-6 exerts impacts on the lungs and intestines, and determining whether there may be a lung-gut axis in patients with PAH should be the next research direction.

In PH, IL-6 can activate JAK/STAT and further activate the MAPK, PI3K/AKT, NF-κB and BMP downstream pathways. It has also been found that IL-6 has a protective effect on intimal remodeling of Schistosoma-induced PH through upregulation of the IL-6/STAT3/NFATc2 pathway (100). Therefore, IL-6 can activate a variety of downstream pathways and produce a variety of biological effects through the JAK/STAT pathway. In addition, there are series between various signaling pathways, such as the activated PI3K/AKT pathway to activate NF-κB and p38/MAPK to activate NF-κB, and p38/MAPK mediates the inhibition of the BMP pathway and leads to the development of PH (16, 116). IL-6 can directly lead to hypertrophy and fibrosis of cardiac fibroblasts in rats with PH *via* the JAK2/STAT3 signaling pathway (101). This shows that the target organ of IL-6 in PH is not only the lungs, but that the damage to cardiopulmonary function in PH patients occurs simultaneously, and cardiopulmonary interaction may further lead to the worsening of the disease. Many drugs can suppress heart damage and ventricular dysfunction by relieving PH (33, 51, 52, 65). Therefore, finding a drug that has simultaneous therapeutic effects on cardiomyocytes, HPAECs and HPASMCs may be the best way to alleviate PH.

PI3K/AKT has been found to be associated with increased development of PH (123). Ding et al. found that the AKT pathway is inactivated in PASMCs exposed to hypoxia as well as in human and rat pulmonary hypertensive lungs (172). We hypothesize that in the early stage of PH, the body inhibits the PI3K/AKT signaling pathway to prevent activation of its downstream pathway from aggravating disease progression. With the aggravation of hypoxia, inflammation or the action of drugs, inflammatory substances such as IL-6 and upstream signaling pathways activate PI3K/AKT signaling, producing the homogeneous effects of promoting proliferation and inhibiting apoptosis, further aggravating the development of the disease. Atorvastatin upregulates PI3K/AKT to treat PH, while other drugs are mostly used to inhibit the PI3K/AKT pathway (63). This may be due to the different targets that drugs affect through the PI3K/AKT pathway. PI3K/AKT may be one of the pathways involved in the treatment of PH with certain drugs but does not play a major role. CO therapy can alleviate PH through the eNOS pathway (173). Telmisartan, genistein, and tanshinone IIA can cause PI3K/AKT activation and ultimately release NO in the endothelium. Trace amounts of NO help to maintain vascular homeostasis (43, 58, 59). However, the activation of PI3K/AKT can also lead to the activation of NF-κB, mTOR and other pathways and aggravate PH (124). Therefore, the PI3K/AKT pathway plays a dual regulatory role in PH. Further research is needed to determine the dosages of the three drugs and at what stages of PH treatment they should be used.

Other literature has also mentioned the use of CO in the treatment of lung injury (174). However, treatment with toxic CO requires careful determination of therapeutic concentration. Many drug studies involving PH therapy tend to influence HPASMCs and HPAECs through various signaling pathways, inhibit pulmonary artery, remodeling and reduce pulmonary artery pressure. There are few studies focused on how to reduce IL-6 and the expression of various inflammatory factors through anti-inflammatory drugs. Clinical experience suggests that the occurrence of inflammation and arterial smooth muscle and endothelial cell lesions promote each other, and anti-inflammatory therapy itself is conducive to the treatment of PH.

The association of sex hormones and gender imbalance with cardiopulmonary disease has already attracted great attention, especially in the context of PH. The proportion of female patients is higher than that of men, while male patients exhibit greater disease severity (5, 88, 175, 176). The overall decrease in CD68 macrophages in male PH mice and male PH patients caused a decrease in the M1/M2 ratio of monocyte-derived macrophages (macrophage transformation to M2), and M2 polarization-induced proliferation of HPASMCs, vascular fibrosis and remodeling were the causes of exacerbation of the condition in male PH mice patients (88). Male HPAECs are more sensitive to stress and impaired mitochondrial function due to hypoxia, and an increase in the active HMGB1 dimer in the systemic circulation is induced by the nonprogrammed death necrosis of HPASMCs, inducing the development of PH in men (163, 177). These differences may be caused by the protective effects of sex hormones, XY chromosomes, and regulatory T cells (Tregs) on females (178).

As stated above, both hypoxia and HMGB1 can promote the expression of IL-6 in HPASMCs and HPAECs. Hypoxia and HMGB1 can also induce the proliferation and migration of HPASMCs and induce the proliferation of HPAECs but inhibit their migration (162). There is no evidence for gender imbalance in the IL6/HMGB1 signaling pathway, and further research is needed.

## 4 Conclusion

Existing studies have suggested that inflammatory factors are key factors in PH. IL-6 is a multifunctional cytokine that plays a crucial role in the regulation of the immune system. Since IL-6 is one of the most important mediators in the pathogenesis of inflammation in PH, signaling mechanisms targeting IL-6 may become therapeutic targets for this disease.

## Author contributions

W-JX, W-NH, QW and RJ drafted the manuscript and prepared the figures. W-JX, W-NH, Y-LZ and SW participated in the writing of manuscripts and literature collection. W-JX, QW and RJ participated in the drawing of figures. J-XH participated in revision of manuscript. J-XH, W-JX, X-SY and RJ proposed the concept, and revised the manuscript. All authors contributed to the article and approved the submitted version.
